# The IN/OUT assay: a new tool to study ciliogenesis

**DOI:** 10.1186/s13630-016-0044-2

**Published:** 2016-08-04

**Authors:** Ira Kukic, Felix Rivera-Molina, Derek Toomre

**Affiliations:** Department of Cell Biology, Yale School of Medicine, New Haven, CT 06510 USA

**Keywords:** Primary cilia, Ciliogenesis, Light microscopy, Ciliary pocket, Exocytosis

## Abstract

**Background:**

Nearly all cells have a primary cilia on their surface, which functions as a cellular antennae. Primary cilia assembly begins intracellularly and eventually emerges extracellularly. However, current ciliogenesis assays, which detect cilia length and number, do not monitor ciliary stages.

**Methods:**

We developed a new assay that detects antibody access to a fluorescently tagged ciliary transmembrane protein, which revealed three ciliary states: classified as ‘inside,’ ‘outside,’ or ‘partial’ cilia.

**Results:**

Strikingly, most cilia in RPE cells only partially emerged and many others were long and intracellular, which would be indistinguishable by conventional assays. Importantly, these states switch with starvation-induced ciliogenesis and the cilia can emerge both on the dorsal and ventral surface of the cell. Our assay further allows new molecular and functional studies of the ‘ciliary pocket,’ a deep plasma membrane invagination whose function is unclear. Molecularly, we show colocalization of EHD1, Septin 9 and glutamylated tubulin with the ciliary pocket.

**Conclusions:**

Together, the IN/OUT assay is not only a new tool for easy and quantifiable visualization of different ciliary stages, but also allows molecular characterization of intermediate ciliary states.

**Electronic supplementary material:**

The online version of this article (doi:10.1186/s13630-016-0044-2) contains supplementary material, which is available to authorized users.

## Background

Primary cilia are highly conserved, single hair-like organelles that extend from the surface of most human cells [1]. Although the primary cilium was first described in 1898, only within the past two decades it has been shown to sense a vast variety of extracellular stimuli including light, sound, odor, fluid flow and chemical signals [2–6]. Indeed, the primary cilium is analogous to a specialized cellular antenna because multiple signaling pathways converge on it to relay extracellular information [7–10]. Not surprisingly, loss or impairment of the primary cilium leads to a large group of genetic disorders called ciliopathies [11–13] and has been associated with cancer progression and tumorigenesis [10, 14, 15]. Problematically, current assays assume that the mere presence of cilia, regardless of stage, is equivalent to the presence of a cellular antenna. Many studies have investigated the role, function and biogenesis of primary cilia, but have not probed whether the cilium is at an early (i.e., intracellular) or late (i.e., extracellular) stage of ciliogenesis. Here, we demonstrate a new facile assay that can quickly and successfully distinguish between different stages of ciliogenesis. We show that most cilia are not fully emerged and, thus, cannot be assumed to act as cellular antennae.

**Fig. 1:**
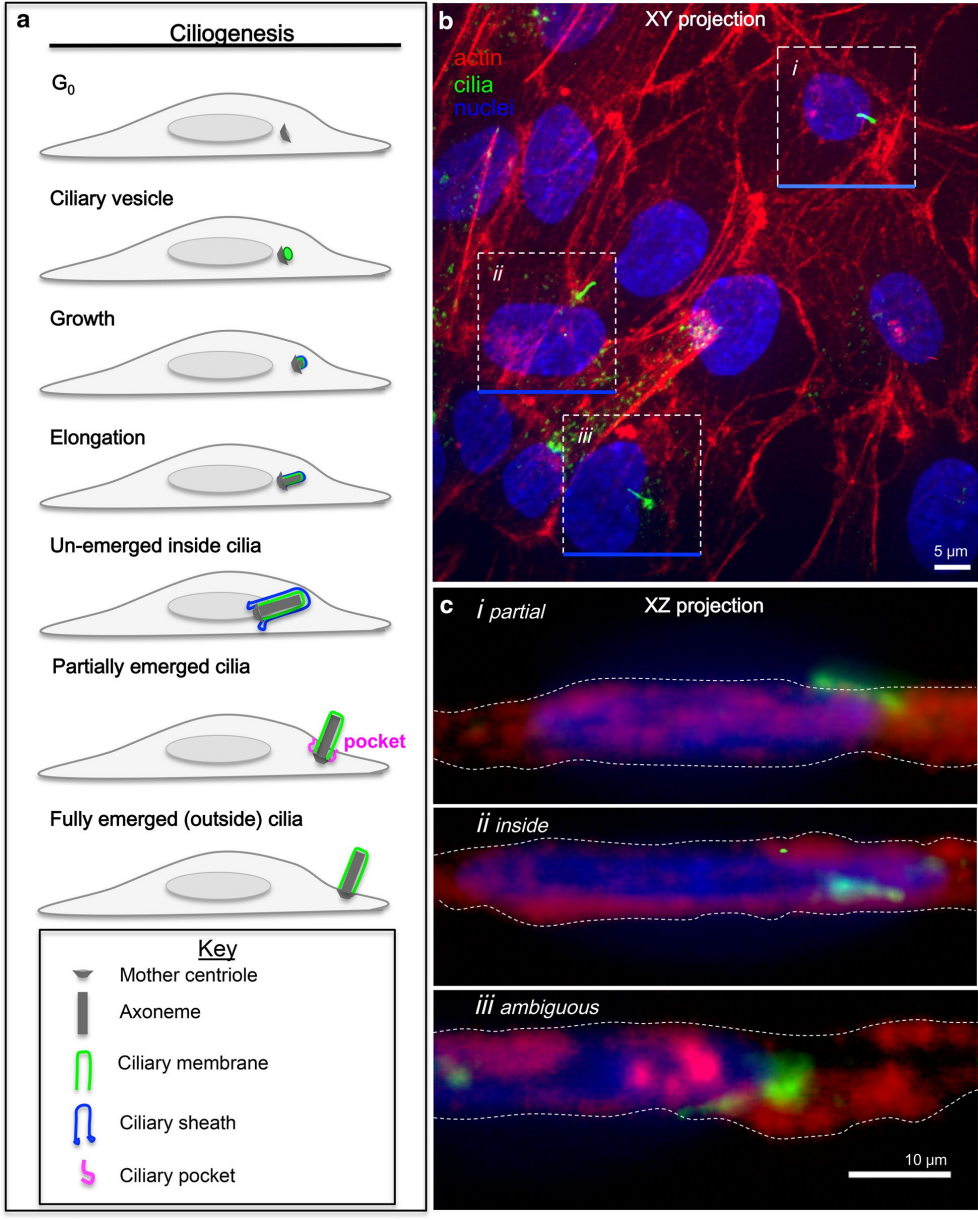
Current ciliogenesis assays are ambiguous. **a** Ciliogenesis model based on Sorokin’s EM data [16–18]. **b** XY projection of htert-RPE1 cells stably expressing pH Smo (*green*) after serum starvation for 48 h and staining for actin (*red*, Alexa Fluor 568 phalloidin) and nuclei (*blue*, Hoechst dye), imaged by SDCM. **c** XZ projections from indicated *boxed areas* in **b**. Projection is of multiple *z-axis* images along the *blue line*. *Dashed lines* in **c** indicate cell outline

Current models of ciliogenesis are founded on seminal transmission electron microscopy (EM) studies by Sergei Sorokin in the 1960s [16–18]. These studies demonstrated that the major route of primary cilia assembly begins with vesicle recruitment to the mother centriole (Fig. 1a). Other vesicles fuse with the small pre-ciliary vesicle [19] which grows and elongates into a large double membrane that surrounds the central microtubule-based axoneme; the membrane associated with the axoneme is called the “ciliary membrane,” which is enveloped by an outer “ciliary sheath” membrane (Fig. 1a) [20]. Ultimately, for the primary cilium to emerge extracellularly, the ciliary sheath fuses with the plasma membrane, which exposes the lumenal face of the ciliary membrane to the extracellular milieu (it is this unique topology that is exploited by the access of a lumenal ciliary probe in our IN/OUT assay). An exception to this ciliogenesis route occurs in polarized epithelial cells [5, 16, 17], where the mother centriole is believed to directly dock onto the plasma membrane. Thus, over the last half century, EM has provided great insights into the unique cellular ultrastructure of cilia.

One of the striking features of ciliogenesis that was revealed by EM is that many cells (except polarized epithelia) have a deep ciliary pocket (Fig. 1a), a poorly characterized structure formed by an invagination of the plasma membrane around the cilium [3, 21]. The function of the ciliary pocket is currently unknown [21], despite being found in many cells including fibroblasts [16, 22], neurons [23–25], keratocytes [26], chondrocytes [27], and oocytes [28]. Analogous ciliary deep pocket invaginations are seen in trypanosomes [29–31], where it is known be a major site of exo-endocyosis and, in spermatids, where it plays an important transient role during spermiogenesis [28, 32, 33]. Yet, the function of the ciliary pocket in most cells remains elusive.

A major bottleneck in studying ciliogenesis is the lack of an easy high-throughput assay to visualize different stages. Although it is possible to visualize cilia via EM in great detail, it is highly improbable that the entire length of an axoneme (~5–10 μm) can be captured within a single 70-nm thick section, as a small tilt will produce an oblique cut. Furthermore, the number of cilia that can be analyzed through this technically demanding and time-intensive approach is very small, making it difficult to investigate stages of ciliogenesis in a rigorous and quantitative manner. Another way to study cilia is by scanning electron microscopy (SEM); however, SEM allows only the emerged portion of cilia to be visualized, and not intracellular portions such as the pocket. By far, the most robust method to study ciliogenesis is immunofluorescence—typically by labeling ciliary proteins such as acetylated tubulin, Smoothened and Arl13b. Although immunofluorescence is amenable to imaging many cilia and quantifying parameters such as cilia prevalence and length, it fails to clearly distinguish between early and later stages of ciliogenesis. We contend that in order to understand the cellular and molecular mechanisms that regulate ciliogenesis, it is necessary to develop a robust, quantitative assay that can unambiguously report different stages of this process.

Here, we describe a new immunofluorescence-based imaging assay in a common model system of retinal pigment epithelial (RPE) cells [19, 34, 35], which successfully identifies different stages of ciliogenesis: intracellular, partially emerged, or fully emerged cilia. Strikingly, despite their appreciable length (~4 μm), up to half of the cilia were intracellular. We validate our assay in proof-of-principle studies and show colocalization of EHD1, Septin 9 and glutamylated tubulin with the “ciliary pocket” region. Overall, the IN/OUT method of labeling cilia allows us to gain better insights into the biogenesis and function of primary cilia, as well as to begin to address the function of the ciliary pocket.

## Methods

### Plasmid construction

To generate the N-terminally pHluorin (pH) tagged Smoothened (Smo) construct, we first generated an hGH signal sequence-pHluorin-hGH (pC4S1-ss-pH-hGH) construct by replacing the 5′XbaI-FM4-FCS-3′SpeI fragment on pC4S1-FM4-FCS-hGH [36] with a 5′XbaI-pHluorin-3′SpeI PCR fragment amplified from Vamp2-pHluorin plasmid (J. Rothman, Yale University). Subsequently, we replaced the 5′SpeI-hGH-3′BamHI fragment of pC4S1-ss-pH-hGH with a PCR amplified minus signal sequence Smo fragment (without the first 35 amino acid) that was cloned by In-Fusion HD directional cloning (Clontech, Inc.) to generate pC4S1-ss-pH-Smo. The ss-pH-Smo fragment was then PCR amplified and cloned by In-Fusion HD into pLVX-puro digested with *Eco*RI and *Bam*HI to generate pLVX-ss-pH-Smo for lentivirus production.

### Tissue cell culture, lentivirus generation and reagents

htert-RPE1 cells (ATCC) were cultured in DMEM/F-12 (Invitrogen) with 10 % FBS (Sigma-Aldrich), 2 mM sodium pyruvate (Invitrogen), 100 U/ml penicillin–streptomycin (Invitrogen), MEM non-essential amino acids (Invitrogen) and supplemented with 50 μg/ml hygromicyn B (Invitrogen) and 10 μg/ml puromycin (Sigma-Aldrich) for selection of pH Smo stably expressing cells. HEK293T cells (Invitrogen) were cultured in DMEM and used for lentivirus production. In brief, HEK293T cells were transfected with 2 μg of pLVX-ss-pH-Smo, 1 μg psPAX2 (Addgene), and 1 μg pMD2.G (Addgene) using Lipofectamine 2000 (Invitrogen). After overnight incubation, the medium was replaced and cells were grown for an additional 48 h. The medium was recovered and centrifuged for 15 min at 1000*g* to remove cell debris and the supernatant was mixed at a 3:1 ratio with Lenti-X concentrator (Takara Bio Inc.) to precipitate and concentrate the virus particles. The remaining pellet was resuspended in 500 μl PBS and 100–150 μl was used to infect RPE cells in the presence of 10 μg/ml polybrene. The following day, the media was replaced and the cells incubated for 24 h before the addition of hygromycin B and puromycin for selection.

The following antibodies were used: GFP (rabbit polyclonal; Invitrogen), GFP (mouse monoclonal; Invitrogen), acetylated α-tubulin (mouse monoclonal; Sigma-Aldrich), Arl13b (mouse monoclonal; NeuroMab), CEP290 (rabbit monoclonal; Bethyl), pericentrin (rabbit polyclonal; Covance), EHD1 (rabbit monoclonal; Abcam), glutamylated tubulin (rabbit polyclonal; Chemicon Int.), Septin 9 (rabbit polyclonal, Sigma-Aldrich), AlexaFluor 568 goat anti-rabbit (Invitrogen) and Atto 647 N goat anti-mouse (Sigma-Aldrich). The following dyes were used: Alexa Fluor 568 phalloidin (Invitrogen) and Hoechst 33342, trihydrochloride, trihydrate (Invitrogen).

### IN/OUT immunofluorescence assay

htert-RPE1 pH Smo cells were plated on glass coverslips and, upon confluency, incubated in serum starvation media containing 0.5 % FBS for 48 h to induce ciliogenesis. Cells were then carefully washed with PBS by slowly submerging (dipping) the coverslip in a beaker containing PBS. Following a 10 min fixation in 4 % paraformaldehyde (PFA) in PBS, cells were washed (as above by dipping) with PBS and blocked in 5 % bovine serum albumin (BSA) for 30 min. Cells were then incubated for 1 h in the first primary antibody against GFP in blocking buffer in a wet chamber to label outside cilia. Following a gentle submerging in blocking buffer, cells were fixed in 4 % PFA for 10 min and then permeabilized with 0.1 % Triton X for 10 min. Then, after a brief dip in blocking buffer, cells were incubated with a second primary antibody against an intracellular ciliary marker (Arl13b, Ac Tub, or Glu Tub, for example) for 1 h in blocking buffer. Following several gentle dipping washes in blocking buffer, cells were incubated with secondary antibodies and Hoechst dye for 30 min, washed again and mounted on a coverslide with Pro-long gold antifade reagent (Invitrogen).

### Microscopy image acquisition and analysis

For 3D imaging, cells were imaged on either a Yokogawa-type Spinning-Disk Confocal Microscope (SDCM, Perkin-Elmer) or on an OMX Structured-Illumination Microscope (SIM). The SDCM is mounted on an inverted microscope base (IX-71, Olympus) equipped with a 1 × 1 Kb electron-multiplying charge-coupled device camera (Hamamatsu Photonics) and a temperature-controlled stage set (in-house). The SDCM is controlled by the Ultraview ERS software (PerkinElmer) and the cells were imaged via a 60 × 1.4 NA oil objective lens with a pixel size of 0.14 µm using 5 solid-state lasers: 405-, 488-, 561-, 594- and 640-nm (Melles Griot). On the SIM, images were acquired using a U-PLANAPO 60X/1.42 PSF, oil immersion objective lens (Olympus, Center Valley, PA, USA) and CoolSNAP HQ^2^ CCD cameras with a pixel size of 0.080 µm (Photometrics, Tucson, AZ, USA) on the OMX version 3 system (Applied Precision) equipped with 488-, 561-, and 642-nm solid-state lasers (Coherent and MPB communications). Samples were illuminated by a coherent scrambled laser light source that passed through a diffraction grating to generate the structured illumination by interference of light orders in the image plane to create a 3D sinusoidal pattern, with lateral stripes approximately 0.270 nm apart. The pattern was shifted laterally through five phases and through three angular rotations of 60° for each Z-section, separated by 0.125 nm. Exposure times were typically between 200 and 500 ms, and the power of each laser was adjusted to achieve optimal intensities of between 2000 and 4000 counts in a raw image of 16-bit dynamic range, at the lowest possible laser power to minimize photo bleaching. Raw images were processed and reconstructed to reveal structures with 100–125 nm resolution [37]. The channels were then aligned in x, y, and rotationally using predetermined shifts as measured using a target lens and the Softworx alignment tool (Applied Precision).

For total internal reflection fluorescence microscopy (TIRFM) and highly inclined and laminated optical sheet (HILO) microscopy, cells were imaged on a microscope (IX-70; Olympus) equipped with 405-, 488-, and 568-nm laser lines, a TIRM condenser (Olympus or custom condenser), a 60 × 1.49 NA TIRF objective (Olympus), an EMCCD camera (iXion887; Andor Technology), a pair of xy Galvo mirrors that are capable of switching from a TIRF light path to wide-field point-scanning FRAP illumination and is controlled with in-house C++ control software (developed by V. Polejaev, Yale University). Calibration of the evanescent field penetration depth was done using ~20 μm silica beads coated with fluorescent rhodamine dye as a reference object of known geometry ([38]; the exact bead diameter was determined by taking a z stack using a PIFOC piezo device [Physik Instrumente]).

Images were analyzed using Volocity software (PerkinElmer) or ImageJ. Specifically, z stacks of images were generated and the frequency and length of the inside and outside portions of cilia were manually measured. XY, XZ and YZ projections were generated using the Volume Viewer plugin for ImageJ. All images were only linearly adjusted for brightness and contrast.

### Statistics

In all cases, except for the cumulative distribution frequency graphs (Fig. 3f; Additional file 6: Figure S6B), statistical significance was calculated using a one-tailed, unpaired Student’s *t* test with * indicating a *p* ≤ 0.05 and ** indicating a *p* ≤ 0.01. Statistical significance in the cumulative distribution frequency graphs (Fig. 3f; Additional file 6: Figure S6B) was tested by a Kolmogorov–Smirnov (KS) test with ** indicating a *p* ≤ 0.001. Data are presented as mean ± s.e.m.

## Results

### Classifying stages of ciliogenesis by current assays is challenging and ambiguous

Current light microscopy-based imaging assays of ciliogenesis use immunofluorescence to localize a ciliary marker [e.g., acetylated tubulin (Ac Tub) or Smoothened (Smo)] to determine ciliary length and frequency. To test whether it was possible to discriminate if the cilia are intracellular or extracellular by 3D confocal imaging, we created a stable cell line where the cilia are labeled with Smo that has an N-terminally tagged pHluorin (pH, a pH-sensitive GFP) in genomically stable retinal pigment epithelial (RPE) cells [34]. We then induced ciliogenesis and demarcated cell outlines using phalloidin (red) to label the actin cortex (Fig. 1b). We used pHluorin as a tag instead of GFP because the former shows less extraneous fluorescence from Smo contained within the more acidic endosomal and Golgi compartments. Figure 1 shows that by looking at the XZ projections, it is possible to see that some cilia protrude from the cell surface (green, Fig. 1c*i*; Additional file 1: Figure S1), while others are entirely intracellular (Fig. 1c*ii)*. However, the intra- or extra-cellular assignment of the majority of cilia was ambiguous, making accurate classification impossible (Fig. 1c*iii*). If cilia are to function as signaling antennae, then they need to be extracellular. Thus, a simple and easy assay that properly distinguishes inside from outside cilia is needed.

### A new assay for imaging different stages of ciliogenesis

**Fig. 2:**
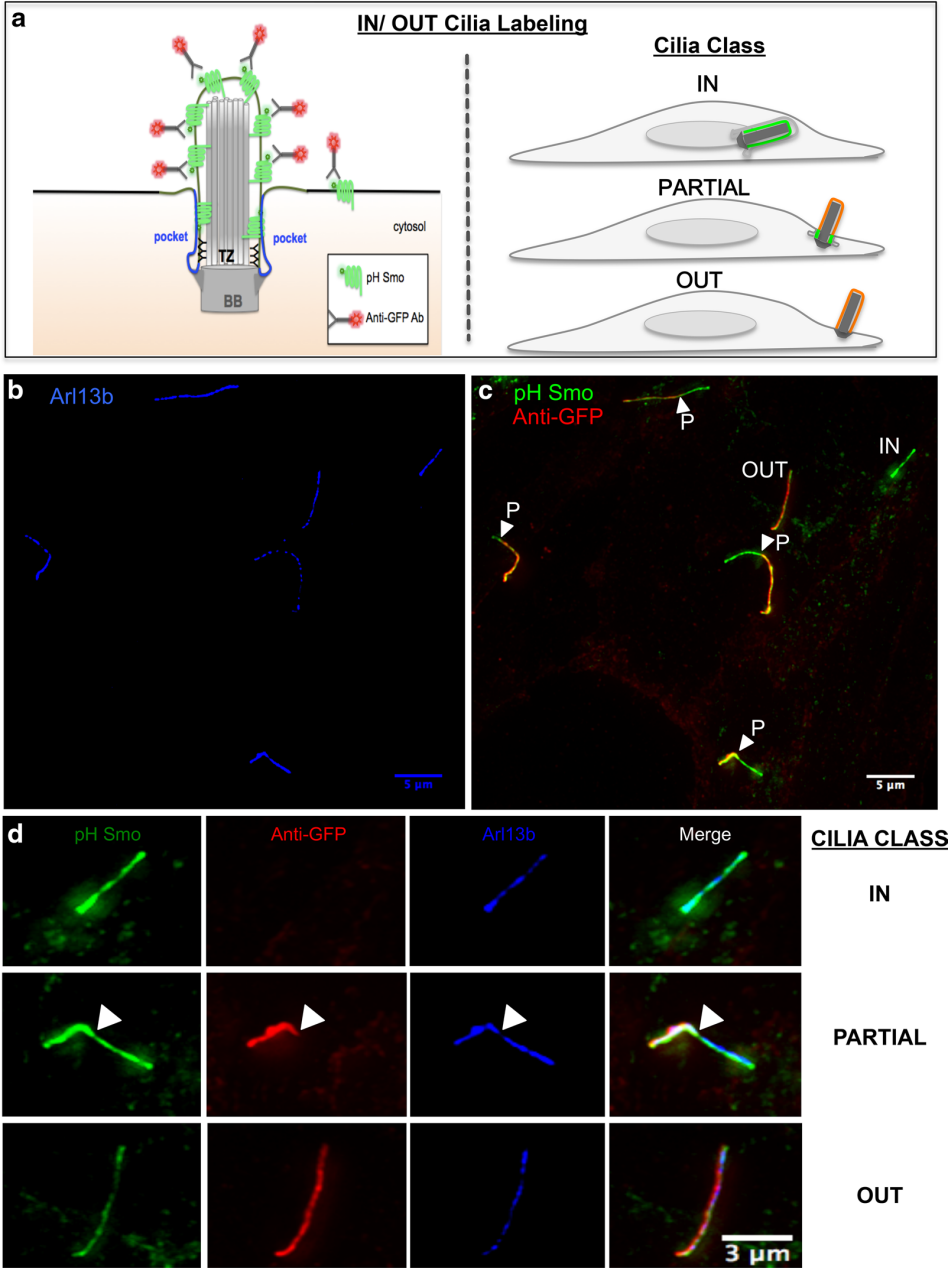
The IN/OUT assay for ciliogenesis. **a** (*left side*) Schematic of the IN/OUT assay. Ciliary membrane is marked by the expression of the pH Smo (*green*) GPCR. Outside/extracellular portions of cilia are labeled by an anti-GFP antibody (*red*) in unpermeabilized cells. Most of the pH Smo localizes to the ciliary membrane but a small fraction remains in the plasma membrane. **a** (*right side*) Diagram illustrating three different ciliary stages. Intracellular cilia (IN) are marked by the lack of anti-GFP signal. Partially extruded cilia (PARTIAL) are marked by the presence of the anti-GFP (*red*) signal on the extracellular side and just pH Smo (*green*) signal on the intracellular (pocket) region of the cilia. Fully emerged cilia lacking pockets (OUT) are marked by a complete colocalization of the *green* and *red* signals (*orange*–*yellow*). **b** htert-RPE1 cells starved for 48 h, fixed, permeabilized and stained with the ciliary marker Arl13b (*blue*). **c** htert-RPE1 cells stably expressing pH Smo (*green*), starved for 48 h, fixed, stained with anti-GFP (*red*) without permeabilization and imaged by structural illumination microscopy (SIM). Note that many different stages of ciliogenesis can be observed within the same field of view including fully inside (IN) and fully outside cilia (OUT) as well as many partially emerged cilia (P). *Arrowheads* indicate the extracellular to intracellular transition on partially emerged cilia. **d** Zoom in images from (**c**) showing the three classes of cilia with Arl13b expression

Since the pHluorin GFP tag was on the N-terminus of Smo and, therefore, exposed to the extracellular environment, we reasoned that we could label extracellular, or emerged cilia by staining non-permeabilized cells with an anti-GFP antibody conjugated to Alexa-568 (Fig. 2a). Using this strategy, we were able to develop a two-color assay in which intracellular cilia are green (Fig. 2a, green); partially emerged cilia are yellow-orange at the surface-exposed region and green at the base. In contrast, fully emerged cilia are completely yellow-orange. This IN/OUT assay allows us to visualize all three different cilia classes (inside, partial and out) within a population of cells that are indistinguishable by current immunofluorescence labeling probes such as Arl13b, even with super-resolved structural illumination microscopy (SIM) (Fig. 2b). Indeed, using the IN/OUT assay in a population of cells, all three classes of cilia emergence (in (IN), partial (P) and out (OUT)) were observed, often within the same field of view (Fig. 2c). As expected, the pH-Smo signal fully overlaps with the bona fide ciliary marker Arl13b [39] staining in intracellular (Fig. 2d, top; Additional file 2: Figure S2), partially emerged (Fig. 2d, middle), and fully emerged cilia (Fig. 2d, bottom).

**Fig. 3:**
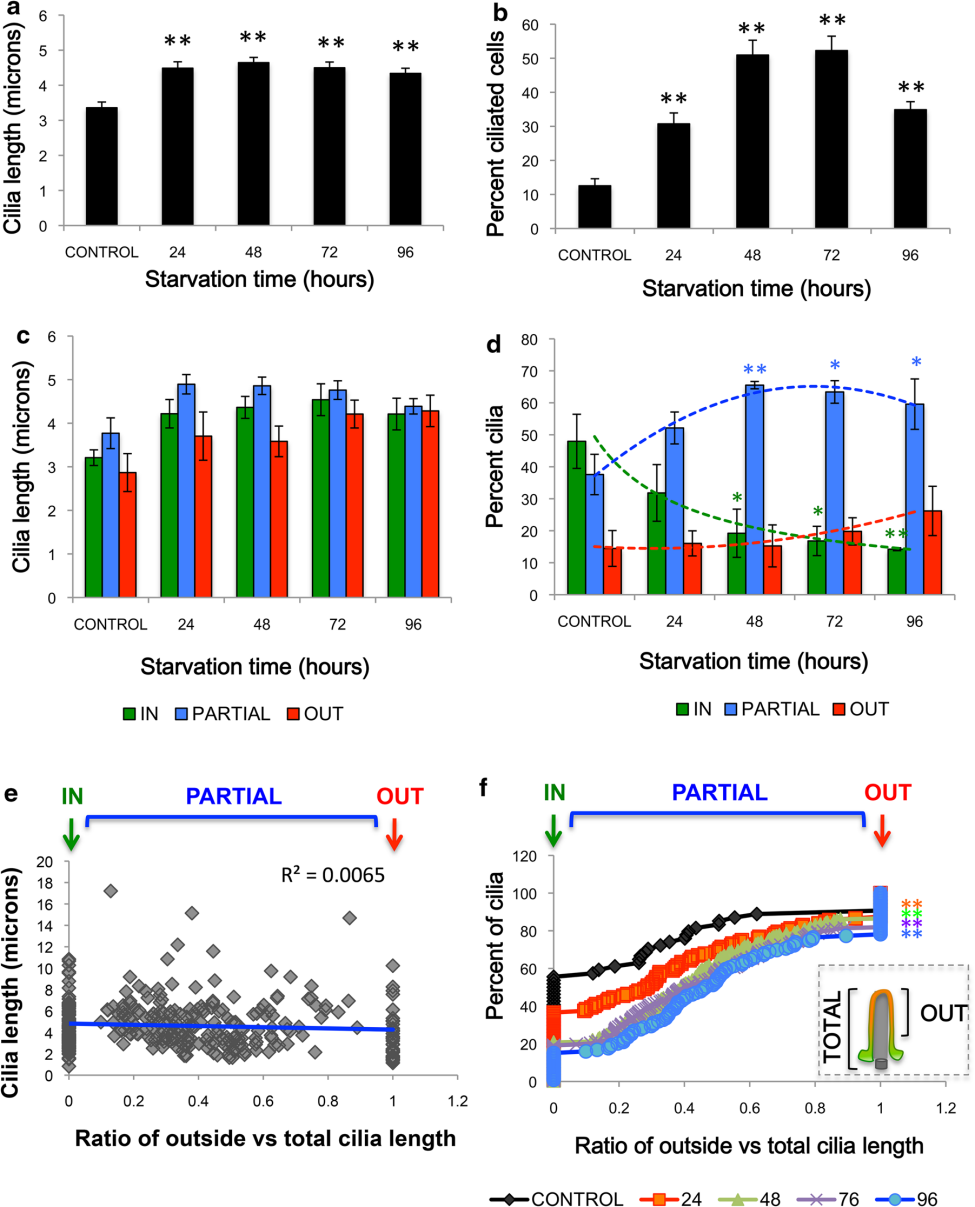
Quantification of stages of ciliogenesis over time. **a** Average cilia length (microns) over serum starvation time (hours) compared to serum control. **b** Average percent of ciliated cells over starvation time compared to serum control. **c** Quantification of average ciliary lengths of in, partial or out cilia. No significant (N.S.) differences were seen between groups. **d** Quantification of the average percent of in, partial or out cilia versus starvation time with* dashed trend lines*. *Asterisk* indicates *p* < 0.05, *Two* *asterisks* indicate *p* < 0.01 by Student’s *t* test compared to serum control. **e** Lack of correlation between cilia length and cilia emergence as measured by the ratio of the length of outside portion of cilia to the total cilia length. Outside cilia have a value of 1 due to complete overlap between outside and ciliary staining, while inside cilia were 0 due to the lack of outside staining. Partial cilia have different ratios depending on the depth of the ciliary pocket. Longer starvation times lead to a greater percent of cilia with higher outside: total ratios compared to serum control. **f** Cumulative distribution frequency of the percent of cilia with outside to total ciliary length ratios over 0–96 h of starvation time. *Two* *asterisks* indicate *p* < 0.01 by KS test compared to serum control

To test if different stages of ciliogenesis can be visualized with the IN/OUT assay, we analyzed cilia over 0–96 h of serum starvation (Fig. 3a–f). We observed a significant increase in total cilium length (4.5 μm ± 0.2, mean ± s.e.m., *p* < 0.001, at 24 h) compared to serum control (3.4 μm ± 0.2, Fig. 3a). This increase in cilia length correlated with a significant increase in the percent of ciliated cells following 24 h serum starvation (30.7 % ± 3.2) compared to serum control (12.5 % ± 2.1, *n* = 15; *p* < 0.001, Fig. 3b). Similarly to what has been reported [19, 40, 41], the percent of ciliated cells peaked at 72 h serum starvation (52.3 % ± 4.2, *n* = 15; *p* < 0.001 compared to control, Fig. 3b). We expected that intracellular cilia would be shorter compared to fully emerged cilia, as ciliary length is often used as a metric of ciliogenesis [34, 42–45] but, interestingly, we found that cilia length does not correlate with cilium emergence (Fig. 3e). In fact, intracellular cilia were similar in length (4.2 μm ± 0.4, Fig. 3c) to partially emerged cilia (4.4 μm ± 0.2, Fig. 3c) and fully emerged cilia (4.3 μm ± 0.4, Fig. 3c) after 96 h serum starvation.

Strikingly, the population of different classes of cilia shifted over the induction time. Intracellular cilia decreased from 48.0 % ± 8.5 to 14.2 % ± 0.5 after 96 h serum starvation (*p* < 0.01, Fig. 3d), while fully emerged cilia increased from 14.5 % ± 5.6 to 26.2 % ± 7.7 after 96 h serum starvation (Fig. 3d). Interestingly, the majority of the cilia were partially emerged with an intracellular ciliary pocket. The percent of partial cilia increased from 37.6 % ± 6.3 to 60.0 % ± 7.9 after 96 h serum starvation (*p* < 0.01, Fig. 3d, f).

These data demonstrate that two commonly used parameters of ciliogenesis—ciliary length and frequency of ciliated cells—are not reliable metrics for identifying functional cilia. For example, although 24 and 96 h serum starvation conditions both yielded 30 % ciliated cells, with cilia of similar lengths (~4.5 μm), 96 h had half the number of inside cilia compared to 24 h (Fig. 3a, b, d).

**Fig. 4:**
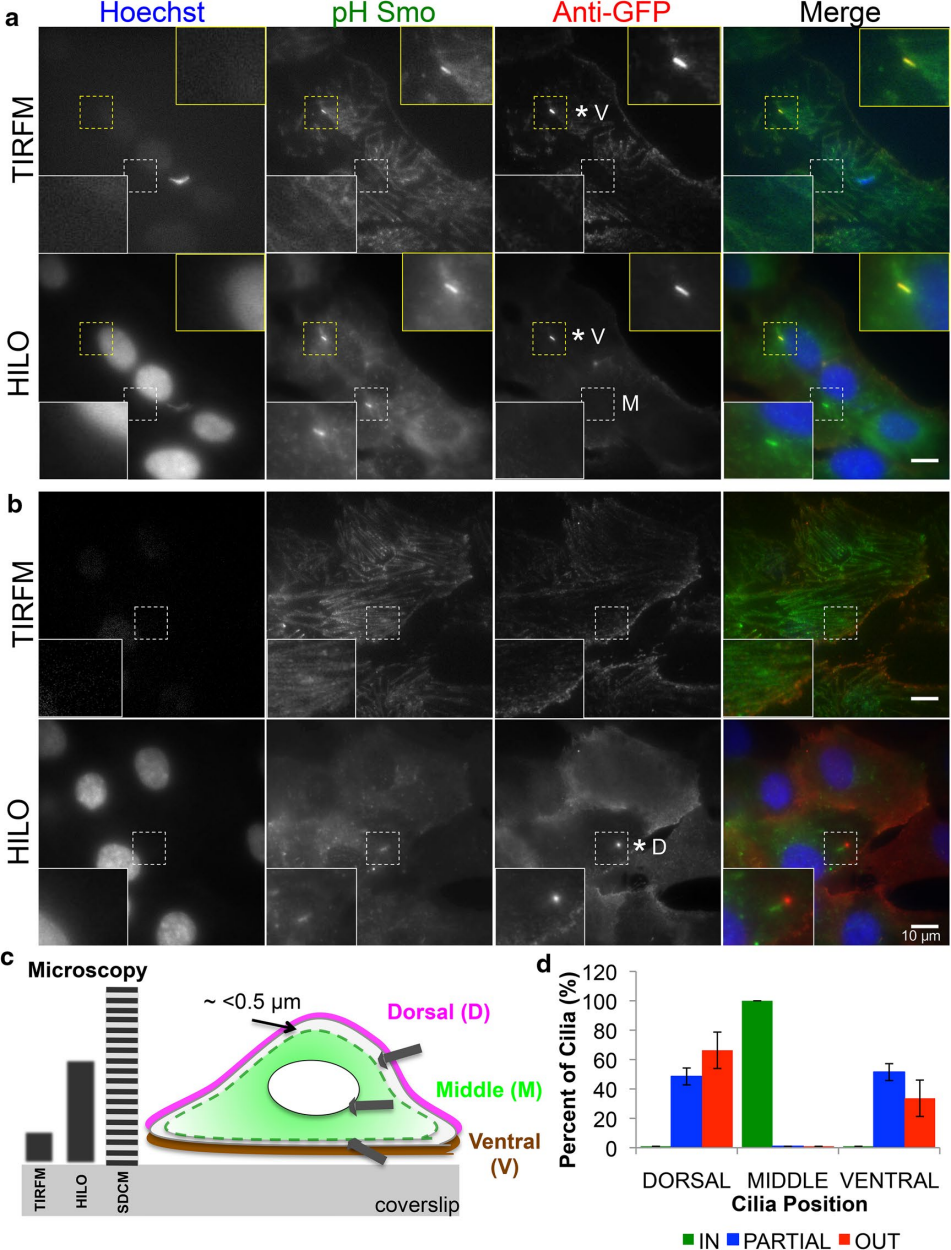
Quantification of dorsal versus ventral cilia emergence. pH Smo expressing cells (*green*) were labeled with the anti-GFP antibody (*red*) and Hoechst dye (*blue*) in fixed, unpermeabilized cells. The cells were then imaged either closer to the coverslip via TIRFM or slightly deeper towards the middle of the cell by HILO within the same field of view. **a** Two cilia in adjacent cells can be seen: one outside near the bottom of the cell seen by TIRFM (*yellow zoom-in box*, marked by *V for ventrally emerged), the other inside within the middle of the cell only visible by HILO (*white zoom-in box*, marked by *M* for middle). **b** A dorsal, partially emerged cilia (marked by *D) not visible by TIRFM, but imaged via HILO. *Asterisk* marks emerged cilia. **c** *Left* schematic of microscopy approaches used and the relative cell depth visualization. *Right* schematic of Dorsal (*D*), Middle (*M*) and Ventral (*V*) cilia emergence classification. Middle cilia are classified as being roughly 0.5 μm away from the membrane. **d** Quantification of the frequencies of cilia emergence relative to cell position from SDCM data

Additionally, contrary to the expectation that most cilia have to translocate to the dorsal side of cells prior to emergence, we observed that cilia can emerge both on the ventral and dorsal cell surface (Additional file 3: Figures S3, Additional file 4: Figures S4, Additional file 5: Figures S5). Using a combination of three different imaging modalities [total internal reflection fluorescence microscopy (TIRFM) [46, 47], highly inclined and laminated optical sheet (HILO) [48], and spinning disk confocal microscopy (SDCM)], we show that approximately half of cilia can emerge and fuse with the ventral surface of cells (Fig. 4a–d). By imaging the same field of view with both TIRFM, which selectively images the lower optical section of cells (<200 nm) near the glass/coverslip interface, and HILO, which images much deeper into the cell, we show that not only does the anti-GFP antibody have access to ventrally emerged cilia close to the coverslip, but also that approximately one-third of the completely outside cilia (33.67 ± 12.39 %) emerge on the ventral surface of cells (Fig. 4a, yellow box). As a corollary, HILO and SDCM imaging shows that inside cilia are not near the dorsal or ventral surface of cells, but are rather, deep in the middle of cells as shown in Fig. 4a (white box). Interestingly, partially emerged cilia did not seem to have a preference for dorsal or ventral emergence, and were observed to emerge ventrally 51.48 ± 5.74 % (Fig. 4b, d) and dorsally 48.52 ± 5.74 % (Fig. 4d). These results are consistent with a model where long inside cilia lacking significant anti-GFP staining (Additional file 5: Figures S5, Additional file 6: Figures S6) can emerge (partially or fully) from deep inside the cell to either the dorsal or ventral surface of cells.

### Molecular characterization of the ciliary pocket

As our IN/OUT assay demonstrates, most cilia (37.5–65.5 % depending on the induction time of ciliogenesis) showed partial anti-GFP staining that had a very distinct demarcation between the stained and unstained regions (Fig. 2d; Additional file 2: Figure S2, arrowheads). We hypothesized that this most likely represented the border between the emerged portion of the cilia and the deep ciliary pocket, which is inaccessible to anti-GFP antibody. As such, we sought to identify cilia markers that colocalize with the pocket.

**Fig. 5:**
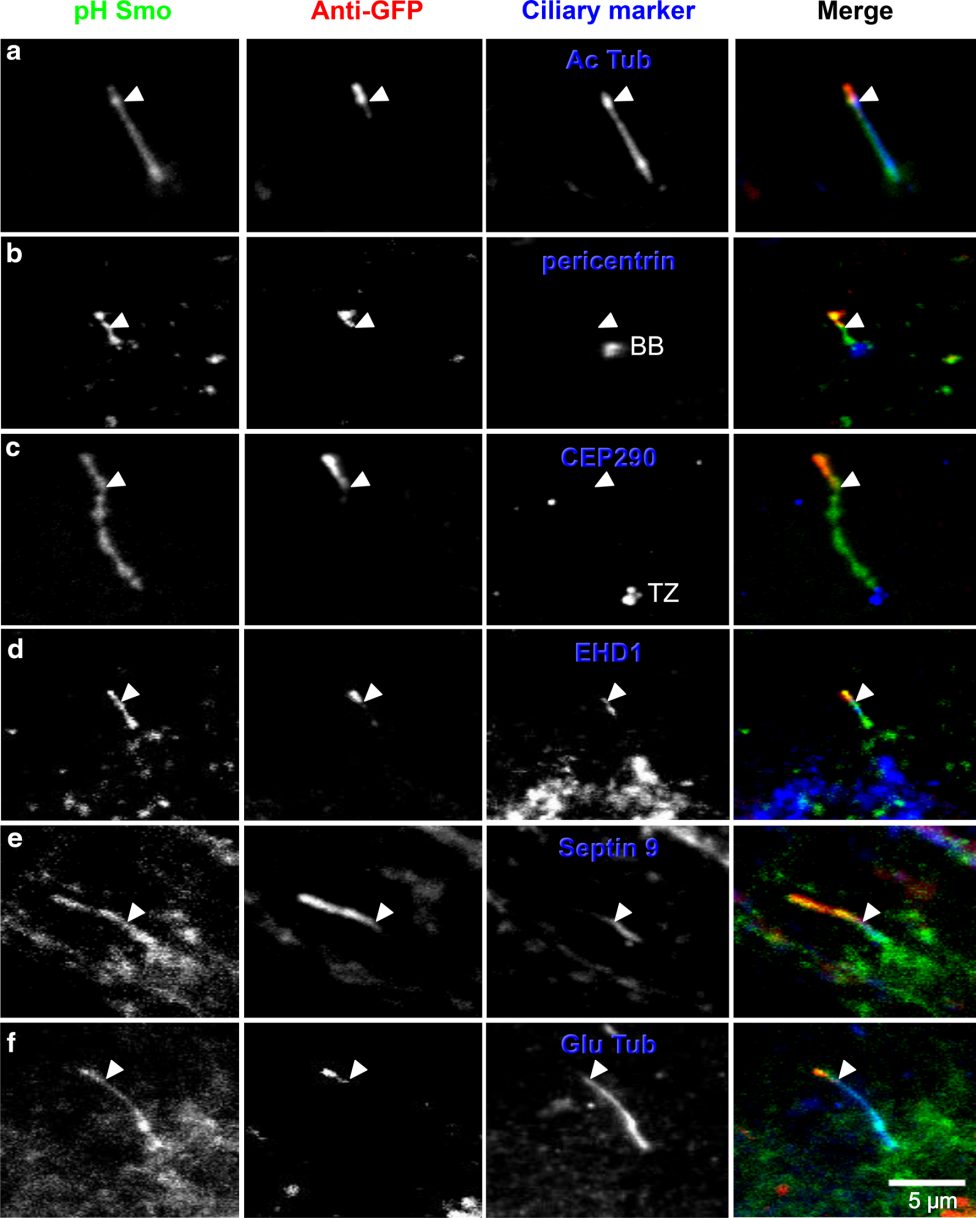
Molecular characterization of the pocket. pH Smo cilia (*green*) were fixed and first labeled with anti-GFP (*red*) in unpermeabilized cells to label the extracellular region (*arrowhead*) and then fixed again, permeabilized and stained with various ciliary markers (*blue*). **a** Acetylated tubulin (Ac Tub) stains the entire length of the ciliary axoneme and does not correlate with anti-GFP staining. **b** The anti-GFP staining ends above the basal body (BB), marked by pericentrin. **c** The anti-GFP staining also ends above the transition zone (TZ), marked by CEP290. **d**–**f** The anti-GFP staining inversely coincides with: EHD1 (**d**), Septin 9 (**e**) and glutamylated tubulin (Glu Tub) (**f**)

To confirm that the anti-GFP staining was not labeling further into the pocket of the cilia towards the transition zone (TZ) or the basal body (BB) (Fig. 2a), we tested the colocalization of various different markers with the outside staining. Firstly, pH-Smo completely colocalized with acetylated tubulin (Ac Tub) staining (Fig. 5a) while the anti-GFP staining did not. Figure 5b shows that anti-GFP staining (red) stopped much higher than the basal body, as marked by pericentrin (BB, blue) [49]. Similarly, the anti-GFP staining (red) did not correlate with the transition zone, as marked by CEP290 (TZ, blue) [50, 51] (Fig. 5c). The demarcation of the anti-GFP staining (red) did, however, abut three proteins which labeled the lower region of the cilia: (1) Eps15 homology domain (EHD) 1, an endosomal membrane shaping protein that was recently shown to control early stages of ciliogenesis [19] (blue, Fig. 5d), (2) a microtubule-binding protein Septin 9 (blue, Fig. 5e), and (3) another ciliary marker that labels the proximal region of the axoneme, glutamylated tubulin (Glu Tub, blue, Fig. 5f).

Septin 9 and Glu Tub were previously shown to localize to primary cilia. Septin 9 is a conserved GTPase that forms hetero-oligomers with other septins, has been shown to interact with microtubules [52] and colocalize along the axoneme of the primary cilia [40]. Interestingly, Septin 9 staining was excluded from the outside portion of the cilia (red, Fig. 5e) and was highly coincident with the region associated with the pocket. Similarly, tubulin glutamylation is a post-translational modification that has been suggested to be important for ciliary function [53–56], and was also excluded from the emerged portion and enriched at the lower half of the axoneme associated with the ciliary pocket (blue, Fig. 5f). Although glutamylated tubulin is associated with the axoneme (and not the ciliary membrane), it is enriched in the proximal region of the axoneme that colocalizes with the pocket region. These data demonstrate that the IN/OUT assay can successfully identify molecular markers of the pocket (EHD1) as well as markers that coincide with the ciliary pocket region (Glu Tub and Septin 9).

## Discussion

An ideal ciliogenesis assay should be robust and easy to perform so that many cells can be studied. EM studies have provided detailed information about the different stages of ciliogenesis; however, due to the low-throughput nature of EM, it has not been clear how abundant each of these stages (inside, partial, or outside) is. Similarly, many studies have relied on immunofluorescence-based screens for factors that regulate ciliary frequency and length without ascertaining whether the cilia are actually inside or outside, an important descriptor of ciliary function. SEM studies, in contrast, have allowed emerged cilia to be visualized, and are better throughput than transmission EM, but cannot study intracellular ciliary components. Rather, SEM only monitors the portion of cilia that extends from the cell surface [57]. Image-based assays in other applications have become standard approaches. For example, the GLUT4-myc translocation assay [58] uses an intracellular GFP tag with an extrafacial myc tag on GLUT4 to monitor GLUT4 translocation to the plasma membrane in response to insulin. This GLUT4 assay is easy, robust, and has become widely used to provide insights into insulin regulation. Similarly, we show that the IN/OUT assay: (1) easily and accurately distinguishes between inside, partially and fully emerged cilia, (2) shows that there is no correlation between ciliary stages and length, (3) shows the frequency of inside/partial/outside cilia emergence relative to the dorsal/middle/ventral region of the cell and (4) demonstrates that specific molecular markers can be assigned to different regions of the cilia. Specifically, our assay provides quantifiable evidence that cilia develop intracellularly, transition through intermediate stages, and some fully emerge on the surface of cells.

As with any tool, there are advantages and disadvantages. One of the disadvantages of the IN/OUT assay is that it depends on the access of an antibody to an exogenous protein, which could be a limitation for cell types that are not easily transfected. However, generating stable cell lines of even hard to transfect cells has become easier with the CRISPR genome editing system [59] and lentiviral constructs. Similarly, the IN/OUT assay cannot readily be used in primary tissue samples, but Septin 9, EHD1 and glutamylated tubulin could potentially be used as endogenous surrogate markers for the pocket or the proximal region of the axoneme associated with the pocket. Despite these limitations, we show the potential of the IN/OUT assay to study ciliogenesis and gain new insight into the function of the ciliary pocket. Future applications include siRNA or drug screens to identify molecules that positively or negatively regulate cilia emergence.

The fact that we observed 4-μm long intracellular cilia raises several questions about the regulation of ciliogenesis. Recent EM data show that pancreatic, breast and brain cancer cells arrest ciliogenesis at an early stage where the internal cilium fails to emerge to the cell surface [10, 60, 61]. These data suggest that the process of primary cilia emergence is highly regulated, but most studies have been unable to address this due to the lack of available tools. Furthermore, here we show that cilia in RPE cells emerged on the bottom (ventral) side of the cell in nearly equal frequency as to the dorsal (top) side. Although ventral cilia emergence has been observed in neuronal stem cells [62], it is not clear whether cilia emerge ventrally in other cell types. Future studies with the IN/OUT assay will provide insights into the progression of ciliary stages and identify the molecular machinery responsible for ciliary emergence.

Data from our studies as well as others [28] have shown that RPE and most other cells have a persistently deep ciliary pocket (several microns long; for a distribution, see Fig. 3f) whose function has remained elusive. The limited research to date suggests that the ciliary pocket is a site of endocytosis [28, 63–66] and that it is involved in basal body (mother centriole) positioning [66, 67] and signal transduction [68]. A morphologically related structure has been described in the connecting cilium of photoreceptors [69–71], the flagellum of spermatids [28, 32, 33], and the flagellar pocket of trypanosomatids (protozoan parasites) [29–31]. However, to our knowledge, there are currently no known reliable markers of the ciliary pocket, making it difficult to study its dynamics and function. As a proof of principle and validation of the utility of the IN/OUT assay, we observed a selective enrichment of specific proteins such as EHD1 at the pocket membrane and Glu Tub and Septin 9 at the proximal region of the axoneme associated with the pocket. Although Septin 9 was reported to bind microtubules along the full length of the axoneme [40], we show that it does not localize to the emerged region of the cilia. Similarly, although glutamylation was present towards the base of the cilia [72] and has been shown to be important for cilia motility [54, 73], we have shown enrichment at the proximal region of the axoneme that is associated with the unemerged portion of the cilia (the ciliary pocket region). These data suggest that perhaps axonemal motility or retrograde transport might be different in the pocket region compared to the rest of the axoneme. These data raise the question of why a deep ciliary pocket might be advantageous and suggest that further studies are needed to fully understand the role of the function of the ciliary pocket, as well as its dynamics and composition.

## Conclusions

Although most cells undergo ciliogenesis, much remains unknown about the process due to the dearth of tools available to study it. Herein, we developed a new ciliogenesis assay to provide more accurate insights into how cilia form and function, as well as show molecular markers of an elusive structure, the ciliary pocket. Future adaptations of the IN/OUT assay could be extended to ciliary disease models to better understand the molecular basis of ciliary perturbations.

## Additional files

10.1186/s13630-016-0044-2 **Figure S1. Cilia localization in 3D space.** pH Smo expressing RPE cells serum were starved for 48 hours, stained with Alexa Fluor 568 phalloidin and Hoechst dyes, and imaged by SDCM. Panels show individual XZ images from the ~ 15 μm z stack starting with 0 μm (bottom of the coverslip) to 13.42 μm (top of the coverslip). Combined XZ images of the stack were used to make the XY and XZ projection images in Fig. 1b and c. *Arrowheads* indicate the base of the cilia while *arrows* indicate the tips of the cilia. For some cilia (**C**), the pH Smo signal emerges above the phalloidin staining, indicating an emerged cilia. Other cilia (**A**), have the pH Smo signal in between phalloidin, indicating an inside cilia. While for most cilia such as in (**B**), the staining is ambiguous.
10.1186/s13630-016-0044-2 **Figure S2. Gallery of in/out ciliary classification.** Representative stages of ciliogenesis. pH Smo RPE cells were induced to form cilia (*green*), stained with anti-GFP (*red*), fixed, permeabilized and stained with Arl13b (*blue*). “IN” cilia lack the anti-GFP (*red*) signal. “PARTIAL” cilia have the outer portion stained with the anti-GFP signal up to the extracellular transition (*arrowhead*). Note the variation in where the transition occurs; some cilia have very deep pockets (top partial cilia), while others have a shallow pocket (bottom partial cilia). “OUT” cilia are fully marked by the anti-GFP signal.
10.1186/s13630-016-0044-2 **Figure S3. 3D visualization of an inside cilia.** **A** XY, XZ and YZ projections of Hoechst (*blue*), pH Smo (*green*), anti-GFP (*red*), and acetylated tubulin, Ac Tub (*purple*) staining of an inside cilium imaged by SDCM. *Dashed line* marks the cell outline. *D* Dorsal cell surface, *V* Ventral cell surface. The anti-GFP signal was linearly increased to see the cell membrane labeling. Merged image is of Hoechst, pH Smo and the anti-GFP signal. *Closed arrowhead* indicates ciliary staining. *Open arrowhead* indicates lack of ciliary staining. **B** Z planes of the merged image of Hoechst, pH Smo and the anti-GFP signal of the same cell. *Arrow* indicates cilia appearance. VENTRAL indicates the ventral anti-GFP surface staining, while DORSAL indicates the dorsal anti-GFP staining.
10.1186/s13630-016-0044-2 **Figure S4. 3D visualization of a ventrally emerged outside cilia.** **A** XY, XZ and YZ projections of Hoechst (*blue*), pH Smo (*green*), anti-GFP (*red*), and acetylated tubulin, Ac Tub (*purple*) staining of a ventrally emerged outside cilium imaged by SDCM. *Dashed line* marks the cell outline. *D* Dorsal cell surface, *V* Ventral cell surface. Merged image is of Hoechst, pH Smo and the anti-GFP signal. *Closed arrowhead* indicates ciliary staining. **B** Z planes of the merged image of Hoechst, pH Smo and the anti-GFP signal of the same cell. *Arrow* indicates cilia appearance. VENTRAL indicates the ventral anti-GFP surface staining, while DORSAL indicates the dorsal anti-GFP staining.
10.1186/s13630-016-0044-2 **Figure S5. 3D visualization of a dorsally emerged partial cilia.** **A** XY, XZ and YZ projections of Hoechst (*blue*), pH Smo (*green*), anti-GFP (*red*), and acetylated tubulin, Ac Tub (*purple*) staining of a ventrally emerged outside cilium imaged by SDCM. *Dashed line* marks the cell outline. *D* Dorsal cell surface, *V* Ventral cell surface. Merged image is of Hoechst, pH Smo and the anti-GFP signal. *Closed arrowhead* indicates ciliary staining. **B** Z planes of the merged image of Hoechst, pH Smo and the anti-GFP signal of the same cell. *Arrow* indicates the emerged cilia tip. VENTRAL indicates the ventral anti-GFP surface staining, while DORSAL indicates the dorsal anti-GFP staining.
10.1186/s13630-016-0044-2 **Figure S6. Quantification of the anti-GFP fluorescence intensity of in, partial and out cilia.** **A** Histogram showing the distribution of the anti-GFP signal from inside, partial and outside cilia from approximately 100 cells. **B** Cumulative distribution frequency of the anti-GFP signal from inside, partial and outside cilia. ** indicates *p* < 0.01 by KS test compared to inside cilia.

## Abbreviations

Ac Tub: acetylated α-tubulin
EM: electron microscopy
GFP: green fluorescent protein
Glu Tub: glutamylated tubulin
HILO: highly inclined and laminated optical sheet
pH: pHluorin
PBS: phosphate-buffered saline
RPE: retinal pigment epithelium
SDCM: spinning-disk confocal microscope
SEM: scanning electron microscope
SIM: structural illumination microscope
Smo: smoothened
TIRFM: total internal reflection fluorescence microscopy
