# Supplementary figures and images for: The IN/OUT assay: a new tool to study ciliogenesis
Source: Cilia. 2016 Aug 4;5:23. doi: 10.1186/s13630-016-0044-2 (PMC4972980; doi:10.1186/s13630-016-0044-2)

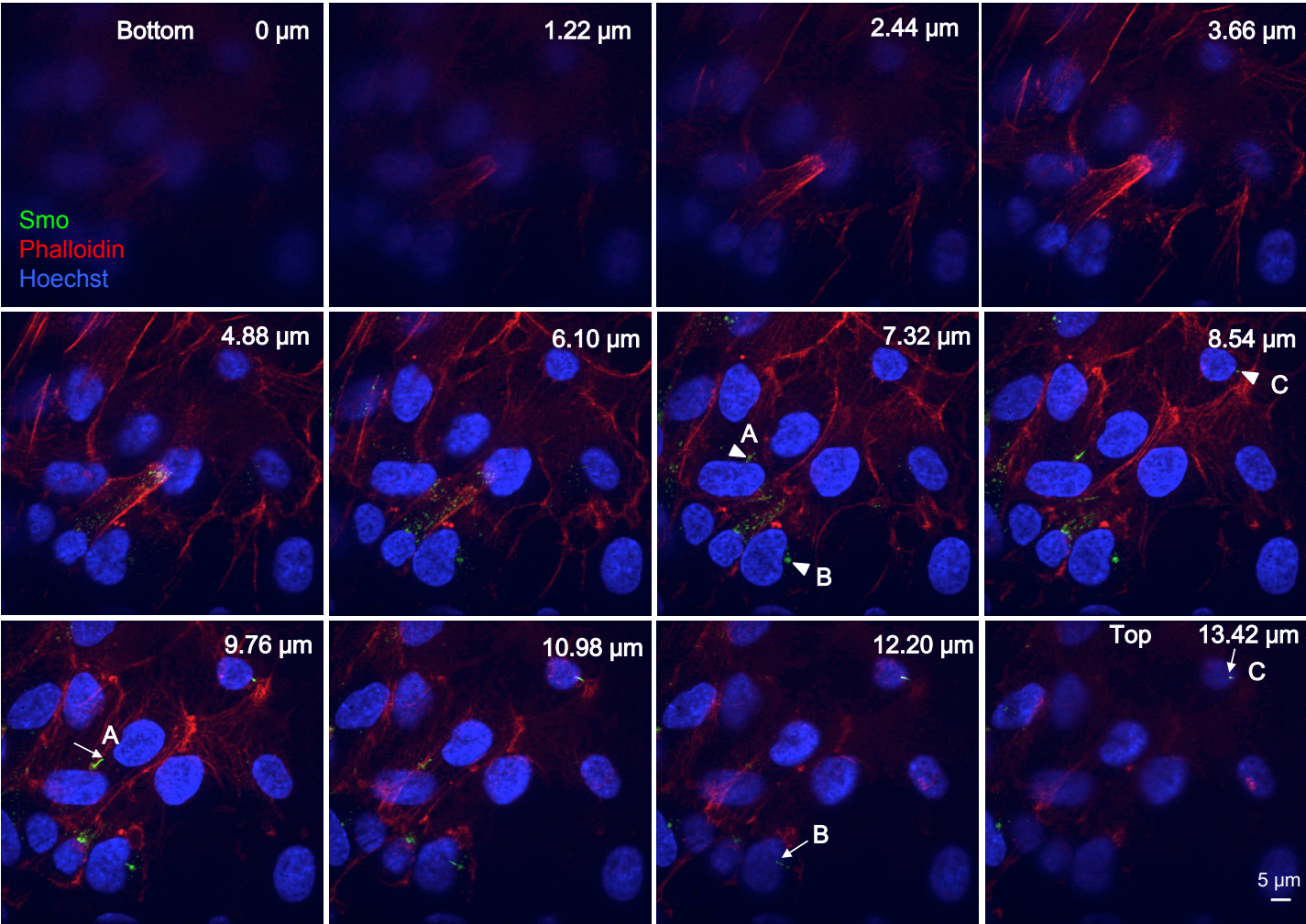

Supplement: Supplementary file 1 — 10.1186/s13630-016-0044-2 Figure S1. Cilia localization in 3D space. pH Smo expressing RPE cells serum were starved for 48 hours, stained with Alexa Fluor 568 phalloidin and Hoechst dyes, and imaged by SDCM. Panels show individual XZ images from the ~ 15 μm z stack starting with 0 μm (bottom of the coverslip) to 13.42 μm (top of the coverslip). Combined XZ images of the stack were used to make the XY and XZ projection images in Fig. 1b and c. Arrowheads indicate the base of the cilia while arrows indicate the tips of the cilia. For some cilia (C), the pH Smo signal emerges above the phalloidin staining, indicating an emerged cilia. Other cilia (A), have the pH Smo signal in between phalloidin, indicating an inside cilia. While for most cilia such as in (B), the staining is ambiguous. [file 13630_2016_44_MOESM1_ESM.pdf]

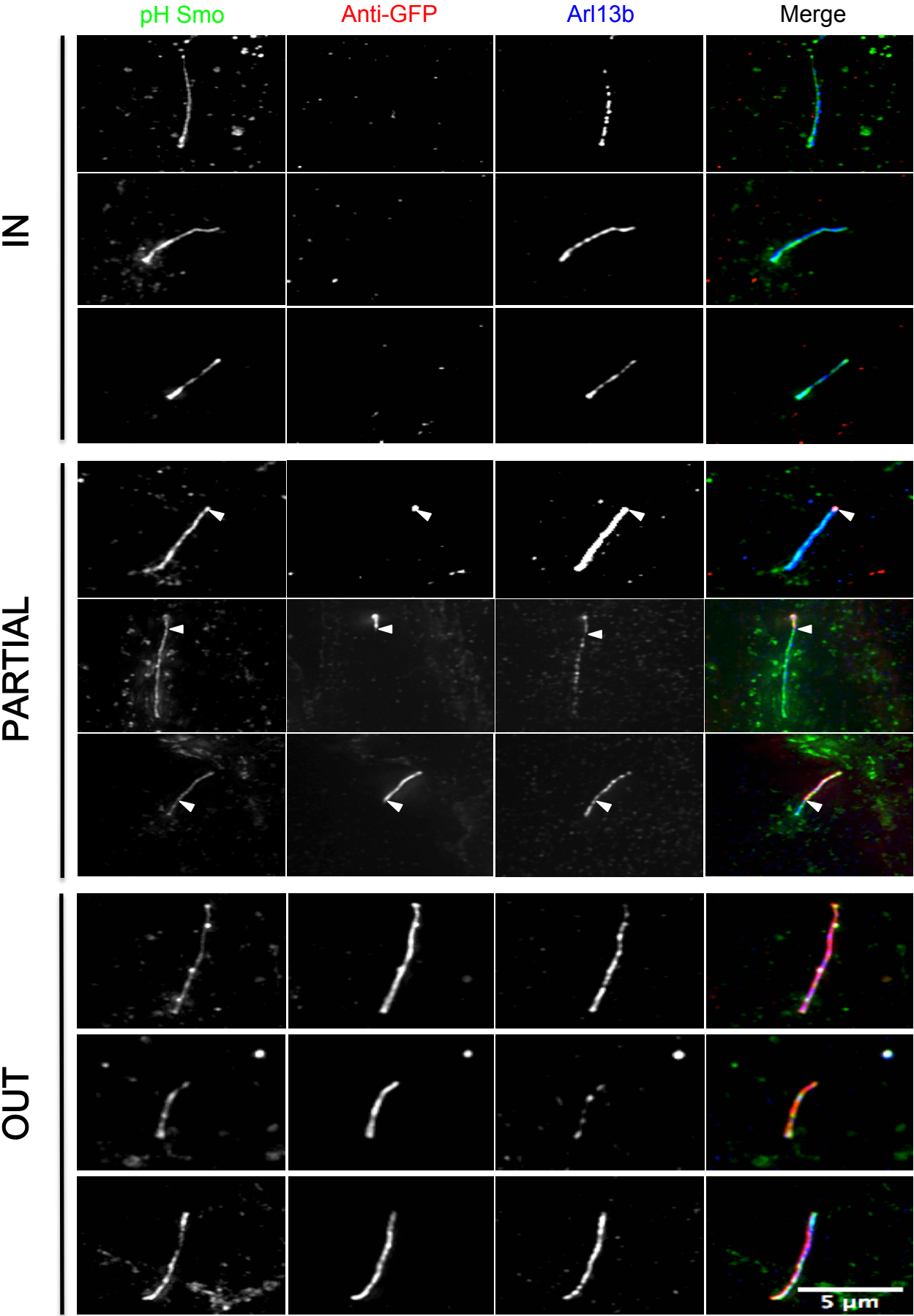

Supplement: Supplementary file 2 — 10.1186/s13630-016-0044-2 Figure S2. Gallery of in/out ciliary classification. Representative stages of ciliogenesis. pH Smo RPE cells were induced to form cilia (green), stained with anti-GFP (red), fixed, permeabilized and stained with Arl13b (blue). “IN” cilia lack the anti-GFP (red) signal. “PARTIAL” cilia have the outer portion stained with the anti-GFP signal up to the extracellular transition (arrowhead). Note the variation in where the transition occurs; some cilia have very deep pockets (top partial cilia), while others have a shallow pocket (bottom partial cilia). “OUT” cilia are fully marked by the anti-GFP signal. [file 13630_2016_44_MOESM2_ESM.pdf]

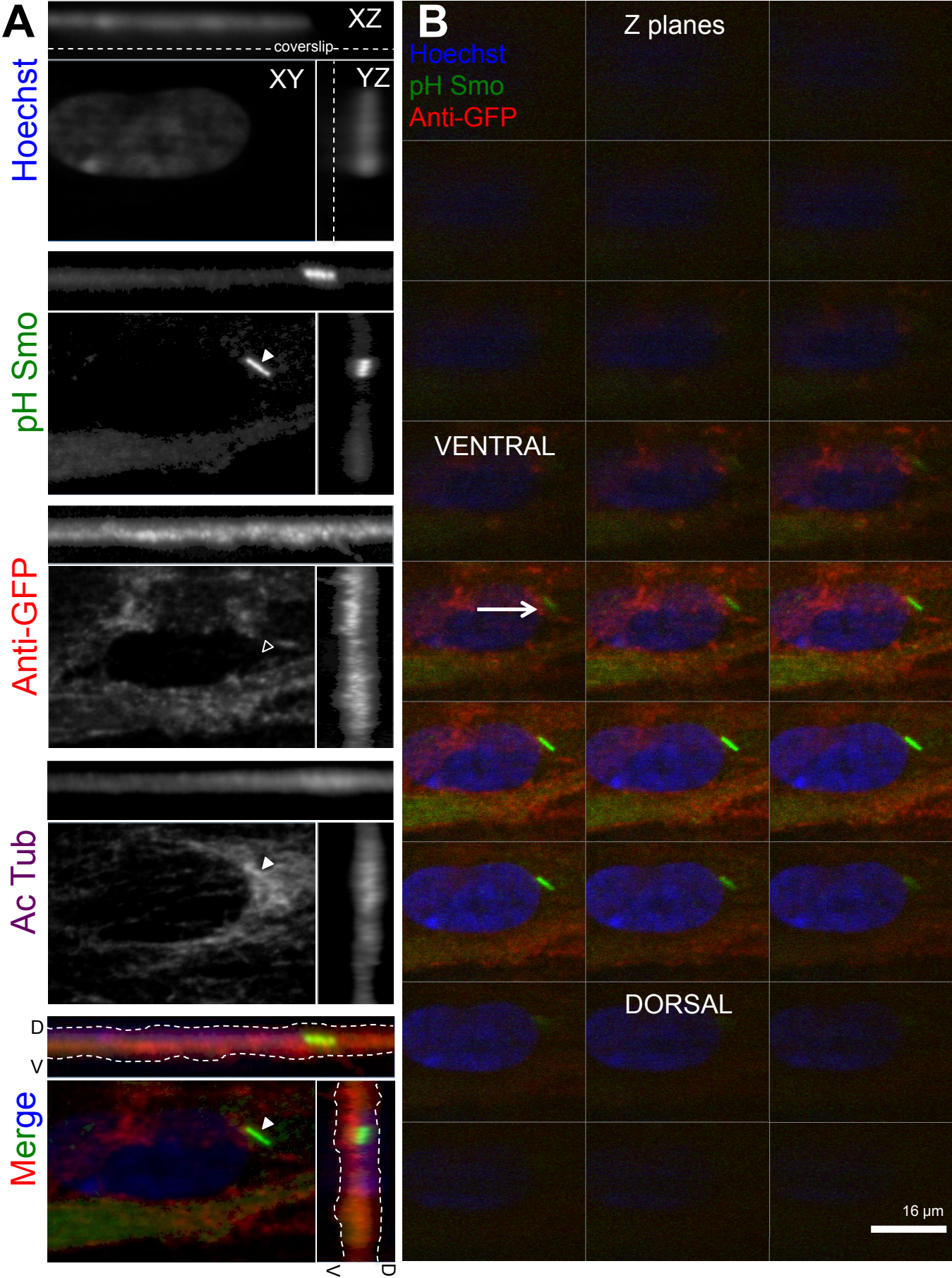

Supplement: Supplementary file 3 — 10.1186/s13630-016-0044-2 Figure S3. 3D visualization of an inside cilia. A XY, XZ and YZ projections of Hoechst (blue), pH Smo (green), anti-GFP (red), and acetylated tubulin, Ac Tub (purple) staining of an inside cilium imaged by SDCM. Dashed line marks the cell outline. D Dorsal cell surface, V Ventral cell surface. The anti-GFP signal was linearly increased to see the cell membrane labeling. Merged image is of Hoechst, pH Smo and the anti-GFP signal. Closed arrowhead indicates ciliary staining. Open arrowhead indicates lack of ciliary staining. B Z planes of the merged image of Hoechst, pH Smo and the anti-GFP signal of the same cell. Arrow indicates cilia appearance. VENTRAL indicates the ventral anti-GFP surface staining, while DORSAL indicates the dorsal anti-GFP staining. [file 13630_2016_44_MOESM3_ESM.pdf]

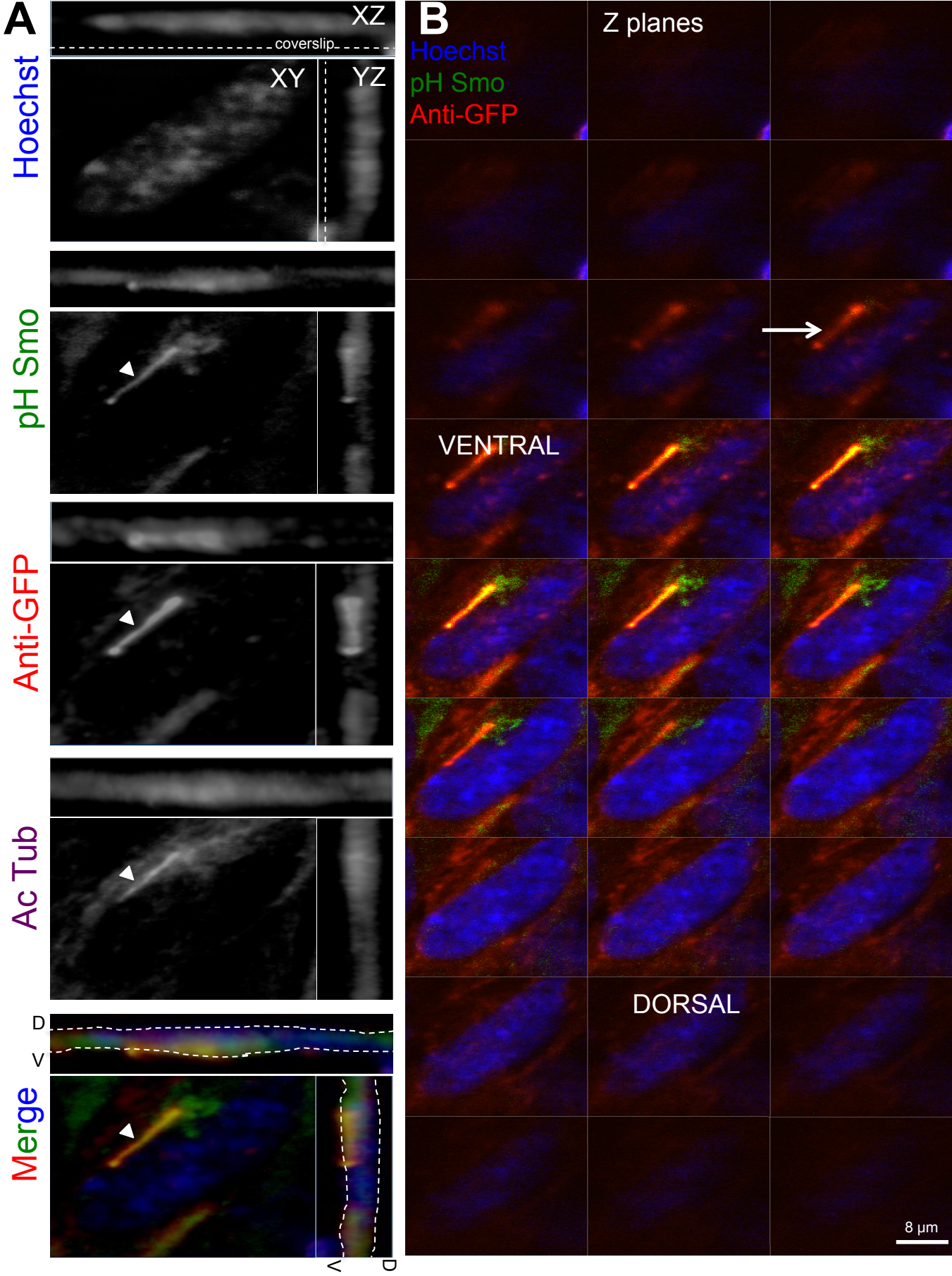

Supplement: Supplementary file 4 — 10.1186/s13630-016-0044-2 Figure S4. 3D visualization of a ventrally emerged outside cilia. A XY, XZ and YZ projections of Hoechst (blue), pH Smo (green), anti-GFP (red), and acetylated tubulin, Ac Tub (purple) staining of a ventrally emerged outside cilium imaged by SDCM. Dashed line marks the cell outline. D Dorsal cell surface, V Ventral cell surface. Merged image is of Hoechst, pH Smo and the anti-GFP signal. Closed arrowhead indicates ciliary staining. B Z planes of the merged image of Hoechst, pH Smo and the anti-GFP signal of the same cell. Arrow indicates cilia appearance. VENTRAL indicates the ventral anti-GFP surface staining, while DORSAL indicates the dorsal anti-GFP staining. [file 13630_2016_44_MOESM4_ESM.pdf]

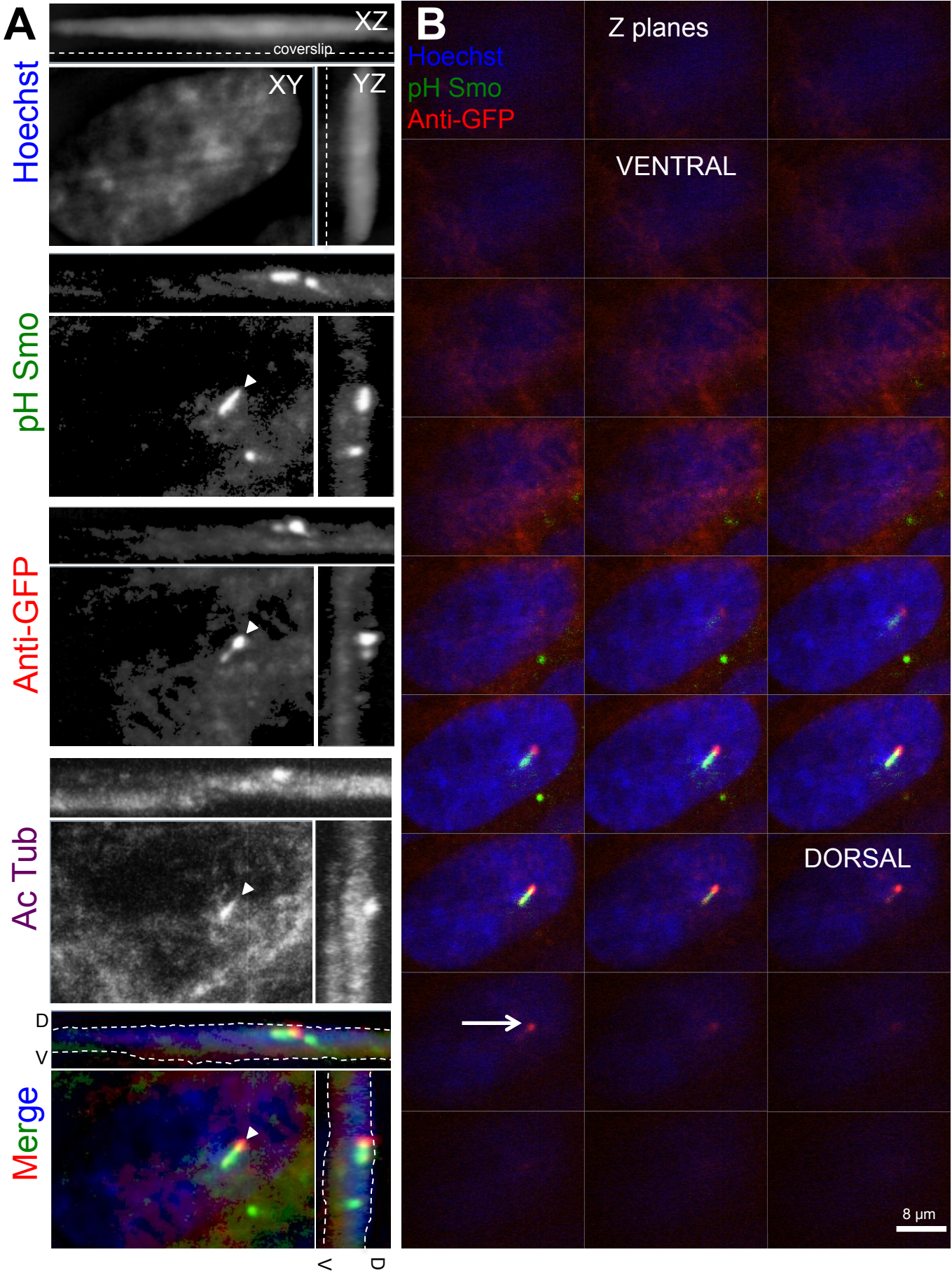

Supplement: Supplementary file 5 — 10.1186/s13630-016-0044-2 Figure S5. 3D visualization of a dorsally emerged partial cilia. A XY, XZ and YZ projections of Hoechst (blue), pH Smo (green), anti-GFP (red), and acetylated tubulin, Ac Tub (purple) staining of a ventrally emerged outside cilium imaged by SDCM. Dashed line marks the cell outline. D Dorsal cell surface, V Ventral cell surface. Merged image is of Hoechst, pH Smo and the anti-GFP signal. Closed arrowhead indicates ciliary staining. B Z planes of the merged image of Hoechst, pH Smo and the anti-GFP signal of the same cell. Arrow indicates the emerged cilia tip. VENTRAL indicates the ventral anti-GFP surface staining, while DORSAL indicates the dorsal anti-GFP staining. [file 13630_2016_44_MOESM5_ESM.pdf]

**A**

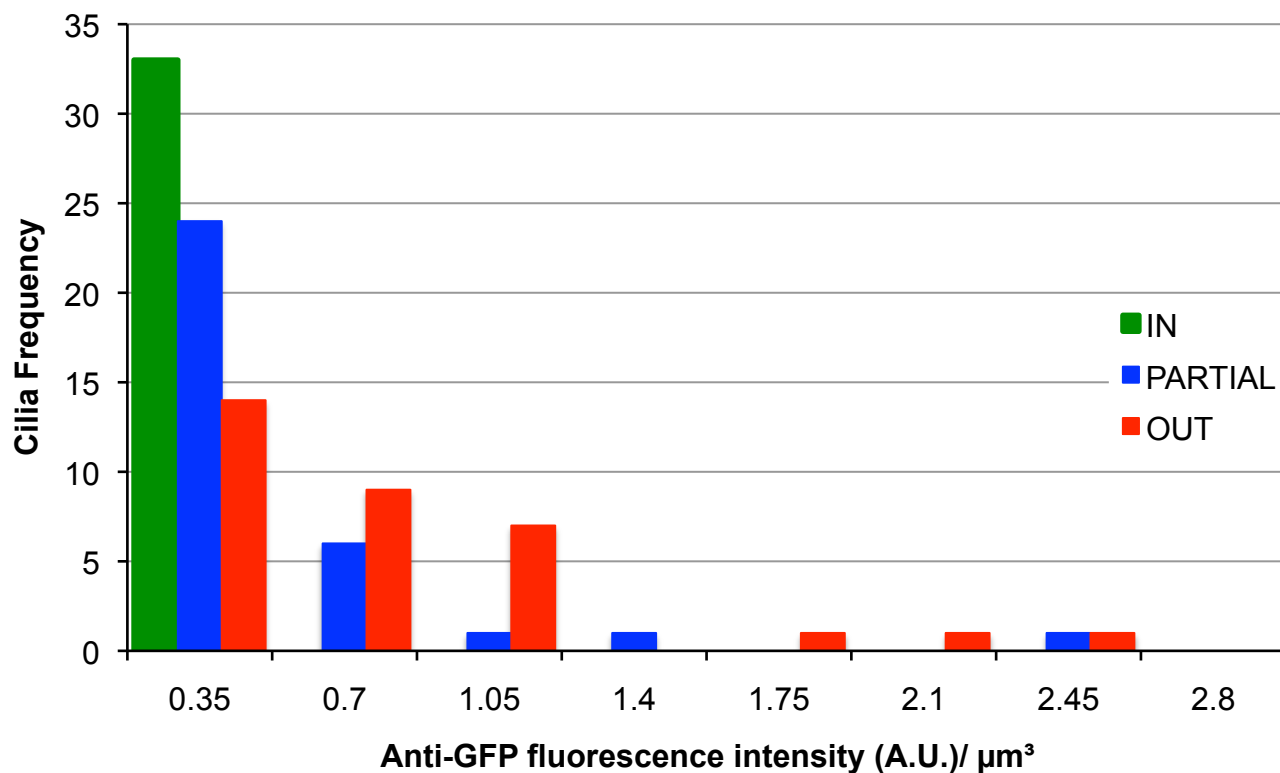

**B**

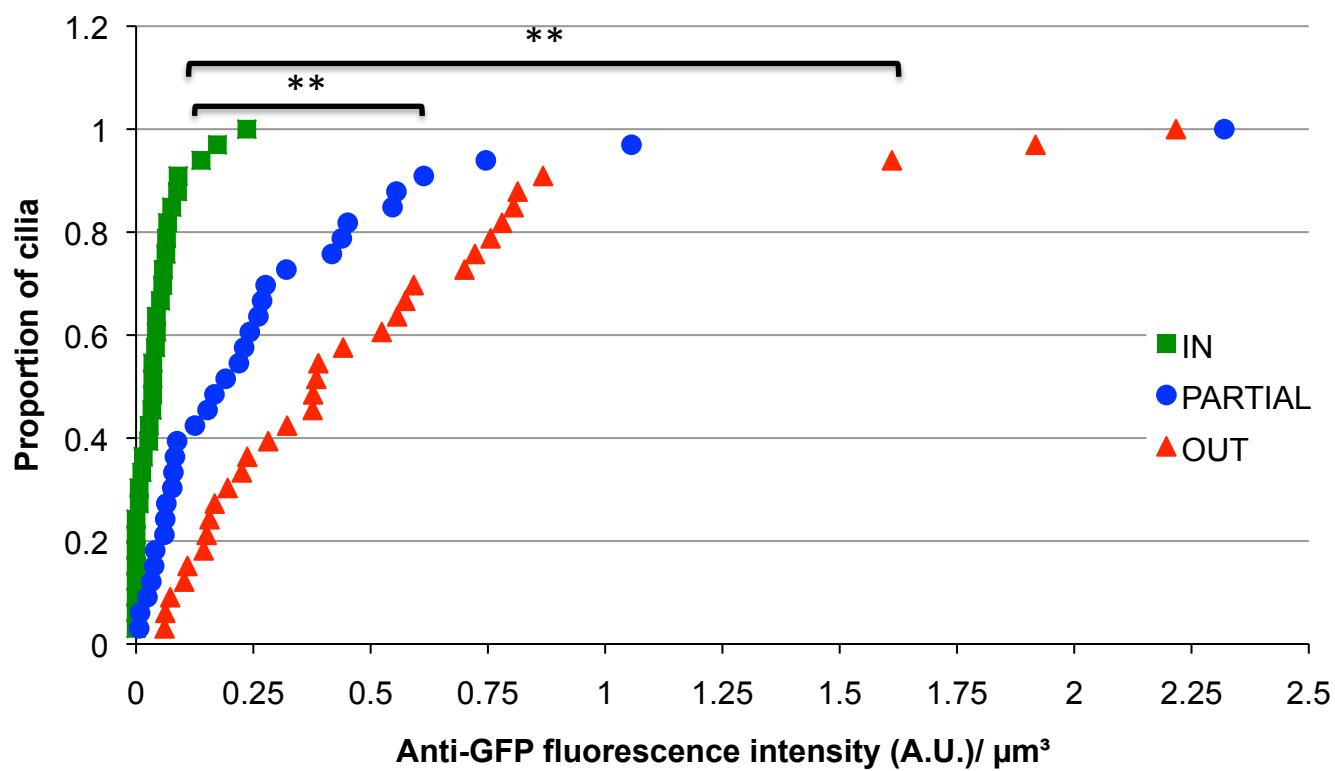

Supplement: Supplementary file 6 — 10.1186/s13630-016-0044-2 Figure S6. Quantification of the anti-GFP fluorescence intensity of in, partial and out cilia. A Histogram showing the distribution of the anti-GFP signal from inside, partial and outside cilia from approximately 100 cells. B Cumulative distribution frequency of the anti-GFP signal from inside, partial and outside cilia. ** indicates p < 0.01 by KS test compared to inside cilia. [file 13630_2016_44_MOESM6_ESM.pdf]
